# The potential value of the neutral comet assay and γH2AX foci assay in assessing the radiosensitivity of carbon beam in human tumor cell lines

**DOI:** 10.2478/raon-2013-0045

**Published:** 2013-07-30

**Authors:** Jin Zhao, Zhong Guo, Hong Zhang, Zhenhua Wang, Lei Song, Jianxiu Ma, Shuyan Pei, Chenjing Wang

**Affiliations:** 1 Medical College of Northwest University for Nationalities, Lanzhou 730030, PR China; 2 Institution of Modern Physics, Chinese Academy of Science, Lanzhou 730000, PR China; 3 School of Life Sciences, Yantai University, Yantai, 264005, PR China

**Keywords:** human tumour cells, carbon ions, X-rays, radiation sensitivity, DNA double strand breaks, γH2AX

## Abstract

**Background:**

Carbon ions (^12^C^6+^) are high linear energy transfer (LET) radiation characterized by higher relative biological effectiveness than low LET radiation. The assessment of tumour radiosensitivity would be particularly useful in optimizing the radiation dose during radiotherapy. The aim of the current study was to evaluate the potential value of the neutral comet assay and γH2AX foci assay in assessing ^12^C^6+^ radiosensitivity of tumour cells.

**Materials and methods:**

The doses of ^12^C^6+^ and X-rays used in the present study were 2 and 4 Gy. The survival fraction, DNA double-strand breaks (DSB) and repair kinetics of DSB were assayed with clonogenic survival, neutral comet assay and γH2AX foci assay in human cervical carcinoma HeLa cells, hepatoma HepG2 cells, and mucoepidermoid carcinoma MEC-1 cells at the time points of 0.5, 4, 16 and 24 h after ^12^C^6+^ and X-rays irradiation.

**Results:**

The survival fraction for 12C6+ irradiation was much more inhibited than for X-rays (p < 0.05) in all three tumour cell lines tested. Substantial amounts of residual damage, assessed by the neutral comet assay, were present after irradiation (p < 0.05). The highest residual damage was observed at 0.5 or 4 h, both for ^12^C^6+^ and X-ray irradiation. However, the residual damage in HeLa and MEC-1 cells was higher for ^12^C^6+^ than X-rays (p < 0.05). The strongest induction of γH2AX foci was observed after 30 min, for all three tumour cell lines (p < 0.01). The franction of γH2AX foci persisted for at least 24 h after ^12^C^6^+ irradiation; in HeLa cells and MEC-1 was higher than after X-ray irradiation (p < 0.05). The correlation coefficients between the clonogenic survival, neutral comet assay and γH2AX foci assay were not statistically significant, except for some tumour cells at individual irradiation doses and types.

**Conclusions:**

Our study demonstrated that the neutral comet assay and γ-H2AX foci assay could be used to assess the radiosensitivity of ^12^C^6+^ in human tumour cells.

## Introduction

Due to their physical and radiobiological properties, high linear energy transfer (LET) radiation qualities are of special interest for tumour therapy. Carbon ions radiotherapy has the potential to broaden the spectrum of primary radiotherapy, as first reports on favourable results for ‘radio-resistant tumours’ like primary renal cell carcinoma have become available and particle irradiation was shown to suppress metastatic potential of cancer cells.^1^,^2^

In order to obtain more insight into this promising strategy some features of the underlying molecular mechanisms inducing the cellular response to carbon ions radiation are currently analyzed. Ionizing radiation causes various DNA lesions such as base damage, DNA-protein cross-links, DNA single-strand breaks and DNA double-strand breaks (DSB). An important prerequisite for a better understanding of such high- LET radiation on the DNA is the mechanistic description of the processing of DSB. It is well-known that the extremely large localized energy deposition and energy ions can lead to particularly complex types of DSB.^3^ Although these effects can lead to cell death, mutations, genomic instability, or carcinogenesis, problems associated with the repair of the high-LET induced DSB are not fully understood.

An estimation of the DNA damage would seem a likely surrogate marker for radiosensitivity because DNA is considered as a primary target of ionizing radiation. The comet assay, which is widely used to quantify DNA damage after exposure to various genotoxic agents, can be a fast and reliable assay system for determining cellular radiosensitivity. Many reporters also have shown that there was a close correlation between γH2AX residual foci and radiosensitivity in some tumour cell lines and γH2AX foci could be used as a sensitive detector of low dose ionizing radiation. The clonogenic assay is the gold standard technique in determining the cellular radiosensitivity. Attempts have been made in the past to correlate DNA damage with the clonogenic survival of the tumour cells using the comet assay and γH2AX assay.^4^,^5^ However, this correlation was not profound, observed in tumour cells with carbon ions (^12^C^6+^) irradiation, suggesting for more studies in this direction for the potential application of the comet assay and γH2AX foci assay in determining the ^12^C^6+^ radiosensitivity of tumour cells.

In the present study, the neutral comet assay and γH2AX foci assay were evaluated for their usefulness in assessing radiosensitivity and the correlation between clonogenic survival, neutral comet assay and γH2AX foci assay in human cervical carcinoma Hela cells, hepatoma HepG2 cells and mucoepidermoid carcinoma MEC-1 after irradiation with ^12^C^6+^ as compared to X-rays. Our studies emphasize the usefulness of the neutral comet assay and γH2AX foci assay in the assessment of the ^12^C^6+^ radiosensitivity of tumour cells.

## Materials and methods

### Cell lines and treatments

Human cervical carcinoma HeLa cell and human hepatoma HepG2 cell were purchased from shanghai institute of biochemistry and cell biology. Human mucoepidermoid carcinoma MEC-1 cell was purchased from School of Stomatology Fourth Military Medical University. The cells were routinely subcultured in Dulbecco’s Modified Eagle Medium (DMEM) (GIBCO, USA), containing 10% newborn calf serum, 100U/mL penicillin, 125 ug/mL streptomycin, and 0.03% glutamine. Exponentially growing cells were seeded at 2×10^4^ cells/100 mm dish and were exposed to different dose of carbon ions (^12^C^6+^) or X-rays. Immediately following irradiation medium was quickly removed and cells were then incubated for various time intervals at 37°C to allow repair.

### Irradiation using carbon ions beam and X-rays

Carbon ions beam was supplied by the Heavy Ion Research Facility in Lanzhou (HIRFL) at the Institute of Modern Physics, Chinese Academy of Sciences (IMP-CAS). Cell exposures were conducted at the therapy terminal of the HIRFL, which had a vertical beam line. Due to the energy degradation by the vacuum window, air gap, Petri dish cover and medium, the energy of the ion beam on cell samples was calculated to be 300 Mev/u, corresponding to a LET of 15 keV/um and the dose rate was adjusted to be about 0.4 Gy/min. The ion beam was calibrated by absolute ionization chamber. The tumour cells were irradiated by plateau of carbon ions LET curve and the dose of scatter off the walls of the plate has been calculated and incorporated into the total dose. The acquisition of the data (preset numbers converted to absorbed dose of particle radiation) was automatically obtained using a microcomputer during irradiation.

Low-LET irradiations were performed using Faxitron Cabinet X-ray System, (Model RX-650, 130kVp, 5mA, USA) operated at 100 kVp. The dose rate was approximately 1.38 Gy/min and the dose used for each irradiation (^12^C^6+^ or X-rays) was 0,2 and 4 Gy. All irradiations were performed once for each dish of cells at room temperature on the same day.

### Clonogenic survival assays

The cells were cultured for 0.5, 4, 16 and 24 h after irradiation and then were washed with phosphate-buffered saline, trypsinized, and counted using a Coulter counter, replated at a density of 5×10^2^ ∼ 3×10^4^ cells in duplicate using 100 mm dishes for cell-survival assays. Plates were stained and colonies were counted two weeks later. Counts from the two plates were averaged, and surviving fraction was calculated as the ratio of the plating efficiency of the treated cells divided by the plating efficiency of the control cells. Experiments were repeated 3–4 times.^6^ The survival fraction was calculated using the following formula:

$$\text{Survival}\;\text{fraction}=\frac{\text{no.of}\;\text{colonies}}{\text{no.of}\;\text{cells}\;\text{plated}\times \left(\text{plating}\;\text{efficiency}/100\right)}$$

### Double-strand break detection using the neutral comet assay

The neutral comet assay was used as described previously.^7^ After the trypsin treatment to produce a single cell suspension, approximately 1.5×10^4^ cells were embedded in 0.75% low-gelling-temperature agarose and rapidly pipetted onto a pre-coated microscope slide. Samples were lysed for 4 h at 50°C in 0.5% SDS, 30 mM EDTA, pH 8.0. After rinsing overnight at room temperature in Tris/borate/EDTA buffer, pH 8.0, samples were electro-phoresed for 25 min at 0.6 V/cm, then stained with propidium iodide. Slides were viewed using a fluorescence microscope with a CCD camera, and 150 individual comet images were analyzed from each sample for tail moment, DNA content, and percentage DNA in tail using CASP software (www.casplab.com). CASP is a tool to image analysis in comet assay. An unlimited number of images can be marked, CASP could load them successively into an “image view” window. Only comets oriented from left (head) to right (tail) can be analyzed correctly. In addition to such parameter as head radius, tail length etc., the program calculates the tail moment (TM) and the Olive tail moment (OTM). Three independent experiments were performed.

### Immunofluorescence microscopy for γH2AX

Immunofluorescent microscopy was conducted essentially as described earlier with some modifications.^8^ Briefly, 2×10^4^ cells were seeded into 35 mm dishes containing a glass cover slip in each well. After irradiation, slides were air-dried, and fixed for 30 min in 2% paraformaldehyde in TBS. Cells were rinsed in TBS, placed in −20°C methanol for 1 min, rinsed, then placed for 20 min in TBS plus 1% bovine serum albumin and 0.2% Tween-20 (TTN) and finally incubated for 2 h with diluted anti-phospho-histone H2AX (Ser-139) mAb (Upstate, Lake Placid, NY) diluted 1:500 in TTN. Slides were washed and incubated with FITC-conjugated anti-mouse goat F (ab′) 2 fragment (DAKO, Carpinteria, CA) diluted 1:200 in TTN for 1 h at room temperature. Slides were rinsed and then immersed in 0.05 mg/mL DAPI for 15 min, rinsed and mounted with cover slips using 10 uL Fluorogard (Bio-Rad) as the antifade mounting medium, and sealed. To prevent bias in selection of cells that display foci, over 800 randomly selected cells were counted. Cells with three or more foci of any size were classified as positive. Experiments were repeated at least three times.

### Statistical analysis

SPSS version 13.0 software program was employed for the statistical analysis. Data were expressed as mean ± standard error (SD). A two-tailed Student’s t-test was also performed to compare the differences between 2 groups. The significance of the correlation coefficient was also calculated. A value of P < 0.05 was considered statistically significant.

## Results

### Growth dynamics of colony survival assay

**FIGURE 1.**
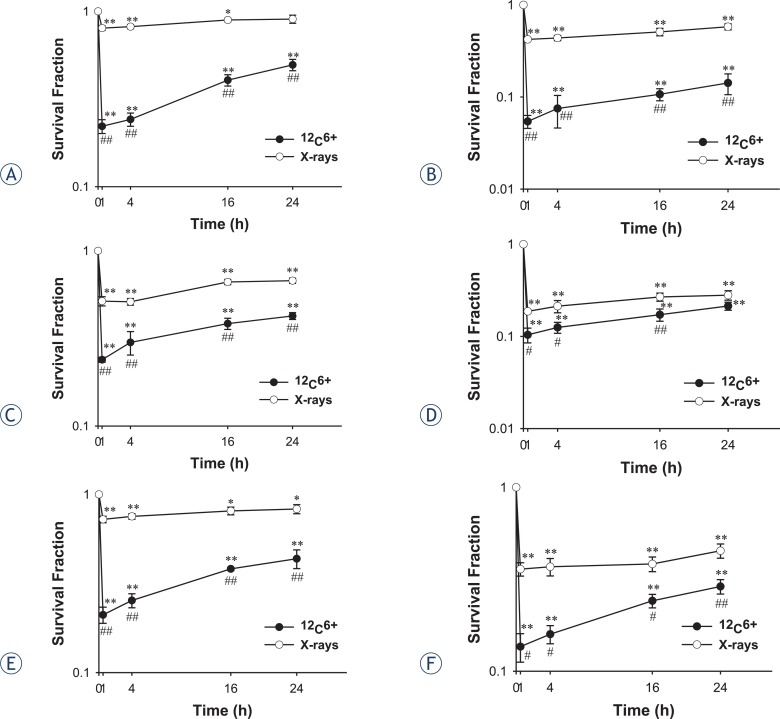
A survival curve for the Hela,HepG2 and MEC-1 cell lines, as determined by clonogenic assay. Exponentially growing cells were plated and irradiated, the cells were taken at the indicated time intervals after irradiation of ^12^C^6+^ and X-rays and a clonogenic assay was performed. The means and SD are shown for three independent experiments with 4 replicates in each experiment. Untreated cells served as a control. After incubation for two weeks, colonies with cells greater than 50 were counted (**A.** Hela-2Gy; **B.** Hela-4Gy; **C.** HepG2-2Gy; **D.** HepG2-4Gy; **E.** MEC-1-2Gy; **F.** MEC-1-4Gy.) *: *P*<0.05 *vs.* 0Gy irradiation; **: *P*<0.01 *vs*. 0Gy irradiation; #: *P*<0.05 *vs*. the same dose x-rays irradiation; ##: *P*<0.01 *vs*. the same dose x-rays irradiation.

Colony assay experiments were performed to compare the differences in terms of the clonogenic growth dynamics of ^12^C^6+^ and X-rays (Figure 1). Clonogenics cells were inactivated immediatedly, but in turn, significantly increased during 24 h after ^12^C^6+^ and X-rays irradiation (p < 0.05). At each time point after irradiation, the increase in the survival for ^12^C^6+^ was much more inhibited rather than for X-rays (p < 0.05), except for HepG2 cell at 4 Gy irradiation (p > 0.05, 24 h). For Hela cells after 2 Gy X-rays irradiation, the survival fraction almost reached the control level at 24 h (p > 0.05), however, the survival fraction for ^12^C^6+^ was still lower (p < 0.01).

### Neutral comet assay

**FIGURE 2.**
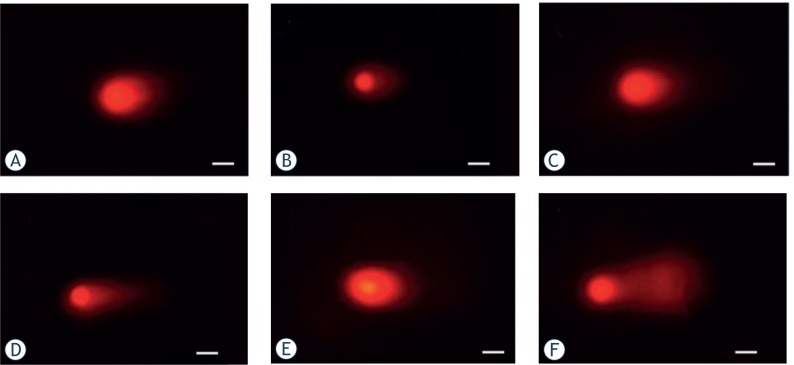
Comet images of DSB detected by neutral comet assay 4 h post-irradiation of ^12^C^6+^ (**A.** Hela-Control; **B.** Hela-4Gy; **C.** HepG2-Control; **D.** HepG2-4Gy; **E.** MEC-1-Control; **F.** MEC-1-4Gy. Scale bar, 50um).

**FIGURE 3.**
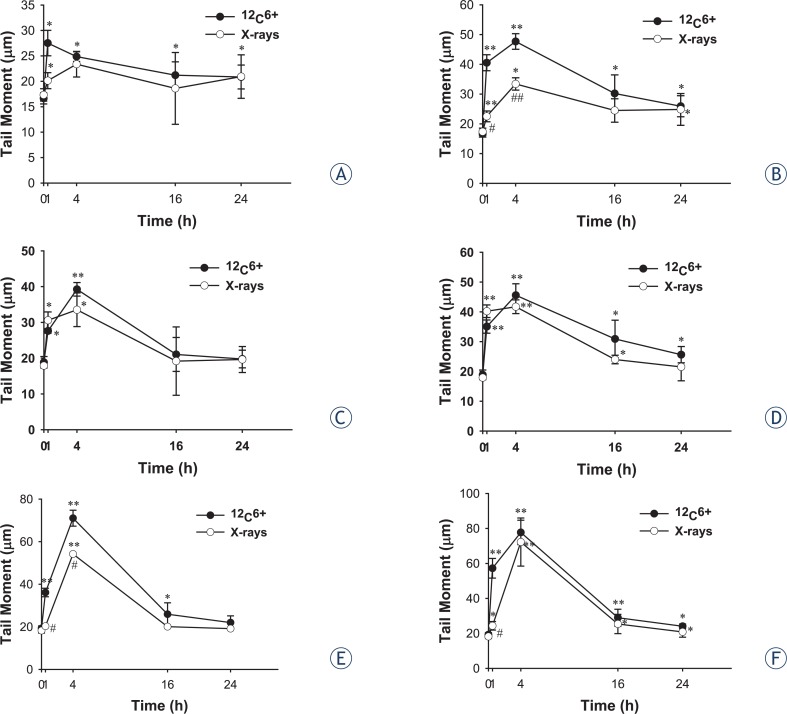
DNA damage induction and repair profiles measured by the neutral comet assay in Hela, HepG2 and MEC-1 cell lines irradiated with 2 and 4 Gy of ^12^C^6+^ and X-rays in vitro. The extent of DNA damage was measured quantitatively by the comet tail moment (TM). Immediately after irradiation, the cell samples were placed at 37° C in a 5% CO2 incubator. The cells were taken at the indicated time intervals after ^12^C^6+^ and X-rays exposure and subjected to the comet assay. The means and SD of TM are shown for three independent experiments with 2 replicates in each experiment. Thirty cells were analyzed for each slide (**A.** Hela-2Gy; **B.** Hela-4Gy; **C.** HepG2-2Gy; **D.** HepG2-4Gy; **E.** MEC-1-2wGy; **F.** MEC-1-4Gy.) *: P<0.05 vs. 0Gy irradiation; **: P<0.01 vs. 0Gy irradiation; #: P<0.05 vs. the same dose x-rays irradiation; ##: P<0.01 vs. the same dose x-rays irradiation.

The neutral comet assays were applied to tumour cell lines exposed to ^12^C^6+^ and X-rays and allowed 0.5, 4, 16 and 24 h for double strand break rejoining and then examined for DSB. Typical comet images were shown at Figure 2. The fraction of residual damage is given in Figure 3. Substantial amounts of residual damage were present for tumour cells after irradiation (*p* < 0.05) and the highest residual damage was almost all shown at 0.5 or 4 h either for ^12^C^6+^ or X-rays. After 0.5 or 4 h of irradiation, the residual damages, contained in HeLa cells and MEC-1, was higher for ^12^C^6+^ than for X-rays (*p* < 0.05).

### Immunofluorescence staining of phosphorylated H2AX foci

**FIGURE 4.**
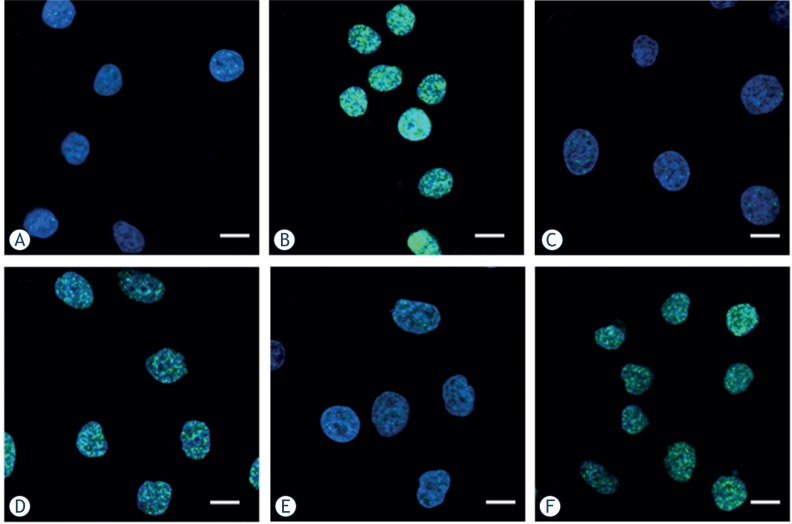
Digitized images of γH2AX foci in Hela, HepG2 and MEC-1 cell lines. After exposure to 4 Gy ^12^C^6+^ followed incubated for 30 min, cells were grown and irradiated on cover slips. DNA was stained with DAPI and γH2AX was detected using an Alexa488- conjugated secondary antibody after staining using a monoclonal anti-γH2AX antibody. (**A.** Hela-Control; **B.** Hela-4Gy; **C.** HepG2-Control; **D.** HepG2-4Gy; **E.** MEC-1- Control; **F.** MEC-1-4Gy. Scale bar, 15um)

**FIGURE 5.**
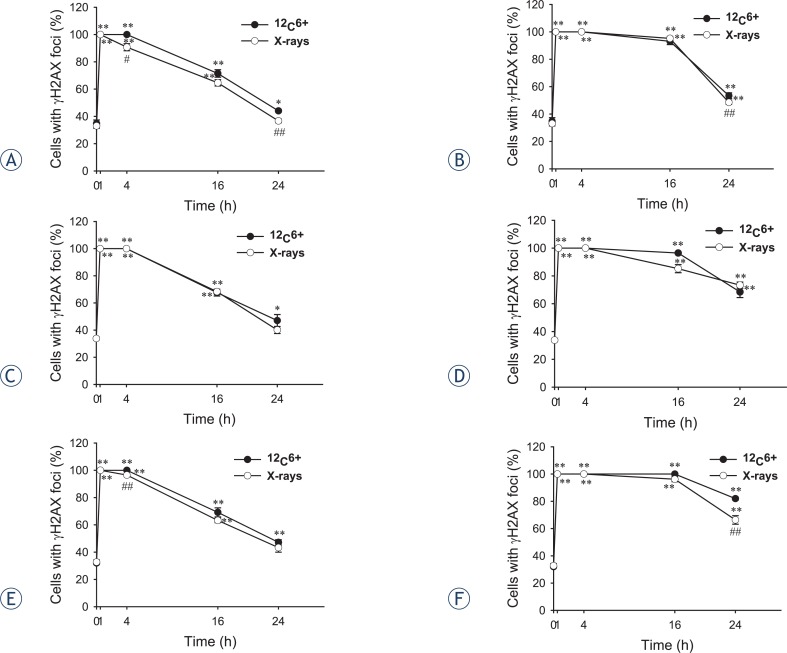
The percentage of γH2AX foci of Hela, HepG2 and MEC-1 cell lines after exposure to 2 and 4 Gy ^12^C^6+^ and X-rays followed incubation for 0.5, 4, 16 and 24 h in *vitro*. Over 800 randomly selected cells were counted. Cells with three or more foci of any size were classified as positive. Results are the means and SD for three experiments (**A.** Hela-2Gy; **B.** Hela-4Gy; **C.** HepG2-2Gy; **D.** HepG2-4Gy; **E.** MEC-1-2Gy; **F.** MEC-1-4Gy). *: P<0.05 vs. 0Gy irradiation; **: P<0.01 vs. 0Gy irradiation; #: P<0.05 vs. the same dose x-rays irradiation; ##: P<0.01 vs. the same dose x-rays irradiation.

Since DSB are the primary toxic lesions induced by radiation, the number of DSB was scored by examining the foci of phosphorylated H2AX in irradiated cells. γH2AX foci were observed with anti-γH2AX antibodies (green) and the nuclei were stained with DAPI (blue). Typical images of ^12^C^6+^ and X-rays induced γH2AX foci are shown in Figure 4. The formation of γH2AX foci in untreated cells was kept at lower levels and no significantly difference was seen between cell lines and culture times (data not shown). After 30 min of radiation, γH2AX foci, visualized as bright spots, were present in all the cells. The time-dependent induction of γH2AX foci by ^12^C^6+^ and X-rays were counted in all tumour cell lines. It was noted the strongest inductions of γH2AX foci at 0.5 h for all three tumour cell lines (p < 0.01) and then decreased over time. A fraction of γH2AX foci persisted for at least 24 h after ^12^C^6+^ radiation in HeLa cells (2 and 4 Gy) and MEC-1 (4 Gy), in contrast to foci induced by X-rays that had a lower level after 24 h (*p* < 0.05, Figure 5).

### The correlation between the clonogenic survival, neutral comet assay and γH2AX foci in ^12^C^6+^ and X-rays irradiated tumour cells

A negative correlation was observed between the clonogenic survival and tail moment assessed by neutral comet assay. The negative correlation was also shown between the clonogenic survival and γH2AX foci. However, a positive correlation was shown between the tail moment and γH2AX foci. However, the correlation coefficients for the most parameters we used, such as different doses and irradiated types, were not statistically significant (P > 0.05, Tables 1, 2 and 3).

## Discussion

In general, it is believed that the DNA damage induced by high-LET heavy ions radiation is more complex than that by X- or gamma rays and leads to more severe biological consequences including cell death, mutation and transformation.^9^,^10^ This complex DNA damage is also referred to as clustered damage or multiply damaged sites, which was originally introduced by Ward.^11^–^13^ These damages could be generated during the processing period of the initially induced DNA lesions. Ionizing radiation even at low doses such as 1 Gy could induce this kind of damage, and with high-LET radiation, the degree of complexity may increase depending on the LET of the particular radiation.^14^ The clustered DNA damage is thought to be more difficult to repair when compared with simple or isolated DNA damage, probably because of retarded enzymatic activities leading to serious biological consequences.^15^

A substantial number of studies on DSB and its repair in cells exposed to high-LET heavy ions have been reported.^16^,^17^ In general, DSB repair is inhibited as a function of LET (up to 200 keV/μm), and the degree of rejoining correlates with the cell survival. If the rejoining is inefficient, a high number of remaining DSB persist after irradiation, leading to a lower cell survival.

In the present study, radiosensitivities of different tumour cell lines to ^12^C^6+^ and X-rays were established using the clonogenic assay and this was correlated with DSB and repair assessed by the neutral comet assay and γH2AX foci. We selected three tumour cell lines which were of different tissue origins. The different cell types were used to ensure that the assay was able to distinguish the radiosensitivity across different tumour types. In the clonogenic assay, a significantly survival inhibition was shown in ^12^C^6+^ and X-rays irradiation over time (Figure 1). It, therefore, seemed reasonable to conclude that an early significant increase in the survival fraction within 24 h occurred after X-rays irradiation, whereas the increase in survival was inhibited after ^12^C^6+^ irradiation. These results obviously showed that ^12^C^6+^ was more cytotoxic than X-rays.

The comet assay can be used when combined with repair enzymes as damage probes to detect DSB for high-LET.^18^–^20^ This method has been shown to correlate radiosensitivity with residual damage, measured at longer times after irradiation.^21^ The assay requires little specialized equipment other than a microscope and under neutral conditions assesses primarily the repair of DSB. From the DNA damage repair analysis, which was performed by measuring the DSB at different time intervals (0.5, 4, 16 and 24 h) and irradiation types, the differences of DSB depended on repair time and irradiation types. For example, the most severe damage was observed after 0.5 or 4 h repair and the damage decreased with time of repair. At 0.5 or 4 h, the residual damages contained in HeLa and MEC-1 cells were much higher for ^12^C^6+^ compared to X-rays (Figure 3).

The results of the neutral comet assay were also compared with those of clonogenic assay in determining the radiosensitivity of the tumour cell lines for different irradiation types. For the three cell lines, the DNA repair kinetics after ^12^C^6+^ and X-rays irradiation, as measured using the neutral comet assay, failed to strongly correlate with the radiosensitivity of clonogenicity, which is in agreement with some of the earlier reports but not with other studies.^4^,^22^ Though DNA repair capacity is one major determinant of the radiosensitivity of the cell, a comet assay may not be sensitive enough to resolve the smaller differences in the residual DNA damage between the cell lines and irradiation types. Our results also showed that the tail moments of the comets had mostly returned to control by 24 h, whereas the survival fraction had not. The reason was that the higher doses (> 10 Gy) were usually used in comet studies, which was significantly higher than the dose range used for the cell survival.^23^,^24^ For example, pulsed-field gel electrophoresis experiments have established that high LET radiation induces persistent DNA double-strand breaks for doses in the range of 25–80 Gy.^25^,^26^ In the present study the doses of 2 and 4 Gy were used for both clonogenic assay and comet assay. The smaller doses usually produced lesser DSB results in the residual damages after 24h contained in cells almost could not be detected by the comet assay.

In 1998, Bonner’s group identified a site in his-tone molecules, namely H2AX, which is phosphorylated after DSB is induced.^27^ The “so-called” γH2AX assay was introduced to detect DSB by simply counting foci in cells using an antibody of γH2AX.^27^–^30^ γH2AX foci along with other radiation induced foci (RIF) were also used to observe the spatial distribution of the radiation tracks from high-LET heavy ion particles.^31^–^35^ Asaithamby *et al*. indicated that high- LET iron ions induced more clustered DNA damage than low-LET radiation; more colocalized foci from three markers were observed with iron ions.^36^ They also showed that high- LET iron ions led to a significantly higher number of chromosome aberrations per mitotic cell than low-LET radiation.^36^ Although many reporters have shown γH2AX foci could be used as a sensitive detector of low dose ionizing radiation, Tomohiro’s research showed that there was not a close correlation between residual foci and radiosensitivity in some tumour cell lines which showed high expression of endogenous γH2AX foci.^37^ In the present study, we firstly compared the background values of γH2AX in three tumour cell lines. The expression of endogenous γH2AX foci was lower and there was not a significant difference between the three tumour cell lines we used (p > 0.05). We, then, measured foci frequency for up to 24 h and found that a fraction of γH2AX foci persisted for at least 24 h after high LET carbon ions radiation in Hela and MEC-1 cells, in contrast to foci induced by X-rays that had a lower level after 24 h (Figure 5). This confirms the earlier studies that these persistent γH2AX foci as evidence of persistent DSB. Karlsson and Stenerlöw found persistent γH2AX foci for up to 24 h after 1 Gy of high LET nitrogen-ion irradiation in non-proliferating normal human fibroblasts.^38^ Takahashi *et al*. showed a slower decrease in the γH2AX intensity using flow cytometry after exposure to 500 MeV/amu iron ions compared to X-rays irradiation in exponentially growing human AG01522 fibroblasts.^39^ Desai *et al*. also showed a LET dependency for the disappearance of γH2AX clusters in confluent normal human fibroblasts between iron (176 keV/mm) and silicon (54 keV/mm) ions.^40^ A recent study using γH2AX fluorescence indicated that high-LET carbon irradiation contributed significantly to the slow component of γH2AX loss kinetics than X-irradiation (80% after carbon irradiation vs. 47% after X-irradiation) confirming the complex nature of DSB by high-LET radiation.^41^

Some results showed the DNA damage measured right after X-rays exposure could be a better reflection of the radiosensitivity than the residual damage^5^, however, for ^12^C^6+^ irradiation, the results were not clear. For both ^12^C^6+^ and X-rays irradiation, although our results clearly indicated that the γH2AX assay could predict the radiosensitivity of different tumour cells and irradiation types, which was not in line with the results of the clonogenic survival assay and tail moment of comet. This problem can be attributed to that for three tumour cells we used, the highest level of DSB were at 0.5 and 4 h for the comet assay and the strongest inductions of γH2AX foci were at 0.5 h for γH2AX assay, however, the lowest survival fractions should be right after the radiation exposure.

## Conclusions

Our studies showed that the neutral comet assay and γH2AX assay could be used in assessing the radiosensitivity of tumour cells in ^12^C^6+^ irradiation. However, to gain greater confidence in use of these techniques in determining the ^12^C^6+^ radiosensitivity of tumour cells, a larger number of cell lines and validation in a clinical scenario using biopsy samples may be needed.

## Figures and Tables

**TABLE 1.** Correlation coefficient obtained from TM by correlating expression with the SF

**Cell lines**	**X-ray/^12^C^6+^(2Gy)**		**X-ray/^12^C^6+^(4Gy)**	
	
	**r-values**	**P-values**	**r-values**	**P-values**
Hela	−0.447/−0.934	0.55/0.07	−0.270/−0.847	0.73/0.15
HepG2	−0.987/−0.641	**0.01**/0.36	−0.956/−0.758	**0.04**/0.24
MEC-1	−0.377/−0.647	0.62/0.35	−0.402/−0.874	0.60/0.13

**TABLE 2.** Correlation coefficient obtained from γH2AX by correlating expression with the SF

**Cell lines**	**X-ray/^12^C^6+^(2Gy)**		**X-ray/^12^C^6+^(4Gy)**	
	
	**r-values**	**P-values**	**r-values**	**P-values**
Hela	−0.945/−0.987	0.05/**0.01**	−0.894/−0.892	0.11/0.11
HepG2	−0.935/−0.948	0.06/0.05	−0.936/−0.870	0.06/0.13
MEC-1	−0.973/−0.976	**0.03**/**0.02**	−0.989/−0.773	**0.01**/0.23

**TABLE 3.** Correlation coefficient obtained from TM by correlating expression with the γH2AX

**Cell lines**	**X-ray/^12^C^6+^(2Gy)**		**X-ray/^12^C^6+^(4Gy)**	
	
	**r-values**	**P-values**	**r-values**	**P-values**
Hela	0.242/0.872	0.76/0.13	0.232/0.776	0.77/0.22
HepG2	0.892/0.818	0.11/0.18	0.957/0.736	**0.04**/0.26
MEC-1	0.519/0.743	0.47/0.26	0.452/0.607	0.55/0.39

## References

[1] Nomiya T, Tsuji H, Hirasawa N, Kato H, Kamada T, Mizoe J (2008). Carbon ion radiation therapy for primary renal cell carcinoma: initial clinical experience. Int J Radiat Oncol Biol Phys.

[2] Ogata T, Teshima T, Kagawa K, Hishikawa Y, Takahashi Y, Kawaguchi A (2005). Particle irradiation suppresses metastatic potential of cancer cells. Cancer Res.

[3] Prise KM, Folkard M, Newman HC, Michael BD (1994). Effect of radiation quality on lesion complexity in cellular DNA. Int J Radiat Biol.

[4] El-Awady RA, Dikomey E, Dahm-Daphi J (2003). Radiosensitivity of human tumour cells is correlated with the induction but not with the repair of DNA double-strand breaks. Br J Cancer.

[5] Jayakumar S, Bhilwade HN, Pandey BN, Sandur SK, Chaubey RC (2012). The potential value of the neutral comet assay and the expression of genes associated with DNA damage in assessing the radiosensitivity of tumor cells. Mutat Res.

[6] Olive PL, Banáth JP, Durand RE (1996). Development of apoptosis and polyploidy in human lymphoblast cells as a function of position in the cell cycle at the time of irradiation. Radiat Res.

[7] Olive PL, Banáth JP, Durand RE (2012). Heterogeneity in radiation-induced DNA damage and repair in tumor and normal cells measured using the “comet” assay. Radiat Res.

[8] Banuelos CA, Banáth JP, MacPhail SH, Zhao J, Reitsema T, Olive PL (2007). Radiosensitization by the histone deacetylase inhibitor PCI-24781. Clin Cancer Res.

[9] Terato H, Tanaka R, Nakaarai Y, Nohara T, Doi Y, Iwai S (2008). Quantitative analysis of isolated and clustered DNA damage induced by gamma-rays, carbon ion beams, and iron ion beams. J Radiat Res.

[10] Hada M, Georgakilas AG (2008). Formation of clustered DNA damage after high-LET irradiation: a review. J Radiat Res.

[11] Chatterjee A, Holley WR (1992). Biochemical mechanisms and clusters of damage for high-LET radiation. Adv Space Res.

[12] Ward JF (1985). Biochemistry of DNA lesions. Radiat Res Suppl.

[13] Ward JF (1994). The complexity of DNA damage: relevance to biological consequences. Int J Radiat Biol.

[14] Hada M, Sutherland BM (2006). Spectrum of complex DNA damages depends on the incident radiation. Radiat Res.

[15] Georgakilas AG, Bennett PV, Sutherland BM (2002). High efficiency detection of bi-stranded abasic clusters in gammairradiated DNA by putrescine. Nucleic Acids Res.

[16] Rydberg B, Lobrich M, Cooper PK (1994). DNA double-strand breaks induced by high-energy neon and iron ions in human fibroblasts. I. Pulsed-field gel electrophoresis method. Radiat Res.

[17] Taucher-Scholz G, Heilmann J, Kraft G (1996). Induction and rejoining of DNA double-strand breaks in CHO cells after heavy ion irradiation. Adv Space Res.

[18] Ostling O, Johanson KJ (1984). Microelectrophoretic study of radiationinduced DNA damages in individual mammalian cells. Biochem Biophys Res Commun.

[19] Blaisdell JO, Wallace SS (2001). Abortive baseexcision repair of radiation-induced clustered DNA lesions in Escherichia coli. Proc Natl Acad Sci USA.

[20] Holt SM, Georgakilas AG (2007). Detection of complex DNA damage in gammair-radiated acute lymphoblastic leukemia Pre-b NALM-6 cells. Radiat Res.

[21] Olive PL (2009). Impact of the comet assay in radiobiology. Mutat Res.

[22] Nahas SA, Davies R, Fike F, Nakamura K, Du L, Kayali R (2012). Comprehensive profiling of radiosensitive human cell lines with DNA damage response assays identifies the neutral comet assay as a potential surrogate for clonogenic survival. Radiat Res.

[23] Iliakis G, Mehta R, Jackson M (1992). Level of DNA double-strand break rejoining in Chinese hamster xrs-5 cells is dose-dependent: implications for the mechanism of radiosensitivity. Int J Radiat Biol.

[24] Noguchi M, Yu D, Hirayama R, Ninomiya Y, Sekine E, Kubota N (2006). Inhibition of homologous recombination repair in irradiated tumor cells pretreated with Hsp90 inhibitor 17-allylamino-17-demethoxygeldanamycin. Biochem Biophys Res Commun.

[25] Stenerlöw B, Höglund E, Carlsson J, Blomquist E (2000). Rejoining of DNA fragments produced by radiations of different linear energy transfer. Int J Radiat Biol.

[26] Rydberg B, Cooper B, Cooper PK, Holley WR, Chatterjee A (2005). Dose-dependent misrejoining of radiation-induced DNA double-strand breaks in human fibroblasts: experimental and theoretical study for high- and low-LET radiation. Radiat Res.

[27] Rogakou EP, Pilch DR, Orr AH, Ivanova VS, Bonner WM (1998). DNA double-stranded breaks induce histone H2AX phosphorylation on serine 139. J Biol Chem.

[28] Rothkamm K, Lobrich M (2003). Evidence for a lack of DNA double-strand break repair in human cells exposed to very low x-ray doses. Proc Natl Acad Sci USA.

[29] Kato TA, Nagasawa H, Weil MM, Genik PC, Little JB, Bedford JS (2006). Gamma-H2AX foci after low-dose-rate irradiation reveal atm haploinsufficiency in mice. Radiat Res.

[30] Kinner A, Wu W, Staudt C, Iliakis G (2008). Gamma-H2AX in recognition and signaling of DNA double-strand breaks in the context of chromatin. Nucleic Acids Res.

[31] Desai N, Davis E, O’Neill P, Durante M, Cucinotta FA, Wu H (2005). Immunofluorescence detection of clustered gamma-H2AX foci induced by HZE-particle radiation. Radiat Res.

[32] Okayasu R, Okada M, Okabe A, Noguchi M, Takakura K, Takahashi S (2006). Repair of DNA damage induced by accelerated heavy ions in mammalian cells proficient and deficient in the non-homologous endjoining pathway. Radiat Res.

[33] Costes SV, Ponomarev A, Chen JL, Nguyen D, Cucinotta FA, Barcellos-Hoff MH (2007). Image-based modeling reveals dynamic redistribution of DNA damage into nuclear sub-domains. PLoS Comput Biol.

[34] Takahashi A, Yamakawa N, Kirita T, Omori K, Ishioka N, Furusawa Y (2008). DNA damage recognition proteins localize along heavy ion induced tracks in the cell nucleus. J Radiat Res.

[35] Jakob B, Splinter J, Durante M, Taucher- Scholz G (2009). Live cell microscopy analysis of radiation-induced DNA double-strand break motion. Proc Natl Acad Sci USA.

[36] Asaithamby A, Hu B, Chen DJ (2011). Unrepaired clustered DNA lesions induce chromosome breakage in human cells. Proc Natl Acad Sci USA.

[37] Yoshikawa T, Kashino G, Ono K, Watanabe M (2009). Phosphorylated H2AX foci in tumor cells have no correlation with their radiation sensitivities. J Radiat Res.

[38] Karlsson KH, Stenerlöw B (2004). Focus formation of DNA repair proteins in normal and repair-deficient cells irradiated with high-LET ions. Radiat Res.

[39] Takahashi A, Yamakawa N, Kirita T, Omori K, Ishioka N, Furusawa Y (2008). DNA damage recognition proteins localize along heavy ion induced tracks in the cell nucleus. J Radiat Res.

[40] Desai N, Davis E, O’Neill P, Durante M, Cucinotta FA, Wu H (2005). Immunofluorescence detection of clustered gamma-H2AX foci induced by HZE-particle radiation. Radiat Res.

[41] Schmid TE, Dollinger G, Beisker W, Hable V, Greubel C, Auer S (2010). Differences in the kinetics of gamma-H2AX fluorescence decay after exposure to low and high LET radiation. Int J Radiat Biol.

